# Inverting Thermal Degradation (^*i*^*TD*) of Paper Using Chemi- and Physi-Sorbed Modifiers for Templated Material Synthesis

**DOI:** 10.3389/fchem.2018.00338

**Published:** 2018-09-07

**Authors:** Paul R. Gregory, Andrew Martin, Boyce S. Chang, Stephanie Oyola-Reynoso, Jean-Francis Bloch, Martin M. Thuo

**Affiliations:** ^1^Department of Materials Science and Engineering, Iowa State University, Ames, IA, United States; ^2^CNRS, Grenoble INP, Institute of Engineering, 3SR, Université Grenoble Alpes, Grenoble, France

**Keywords:** paper, thermal degradation, ^*i*^*TD* paper, surface modifiers, silanes, Fe_2_Cl_3_

## Abstract

Fibrous cellulosic materials have been used as templates for material synthesis or organization via thermal degradation of the cellulose. Most of these methods, however, fail to exploit fiber organization, in part due to loss of structure with processing. Herein, we demonstrate that chemi- and physi-sorbed modifiers of cellulose alters the thermal degradation mechanism allowing for controlled deposition of oxide and carbon (incomplete combustion) along the original paper fiber network. We demonstrate that the degradation of the cellulose fibers depends on the amount of physisorbed material due, in part, to effect on the propagation of the ignition event. From the distribution of the residual elements and shape of the deposits, we can infer that the thermal degradation process depends on the nature, and concentration, of filler(s) or occluded.

## Introduction

Frugal approaches to organized functional materials synthesis are desired, in part due to the ability to exploit size dependent (nano- to micro-) features of such materials. One approach to achieve this goal is the use of appropriate templates (Xia and Whitesides, 1998) or sacrificial scaffolds (Liu et al., 2011; Tallon and Franks, 2011). Such processes are well known, for example; colloidal crystals/assemblies—to create metallic nanostructures (Jiang et al., 1999; Velev et al., 1999; Kulinowski et al., 2000; Velev and Kaler, 2000; Xu and Goedel, 2005), electrospun fibers (Sakai et al., 2013; Son et al., 2013), secondary bonding networks (Macgillivray et al., 2008), among others. Sacrificial scaffolds are removed post-synthesis using methods like selective dissolution (Li and Sieradzki, 1992; Erlebacher et al., 2001; Ding and Erlebacher, 2003; Ding et al., 2004), subtractive phase changes such as sublimation (Flauder et al., 2014), or breaking secondary bonds (Macgillivray et al., 2008; Bai et al., 2018). Other methods that do not require template removal exploit interface and/or dynamic properties of the assembling materials and include use of scanning probes (Fait et al., 2018) and polymerization induced self-assembly (Zhang et al., 2017). While these methods create well-defined porous nanostructures, they are often complex, labor intensive, expensive or require specialized equipment. A major thermodynamic caveat is that work is required to assemble and disassemble the system, as such, they are overall endergonic processes. Alternatively, exploiting an already porous material as the initial template, that can also undergo controlled exothermic (dissipative) degradation allows the use of precursor moieties that are *in situ* transformed and organized along the template in a coupled oxidation and diffusion limited sintering process.

**Figure 1:**
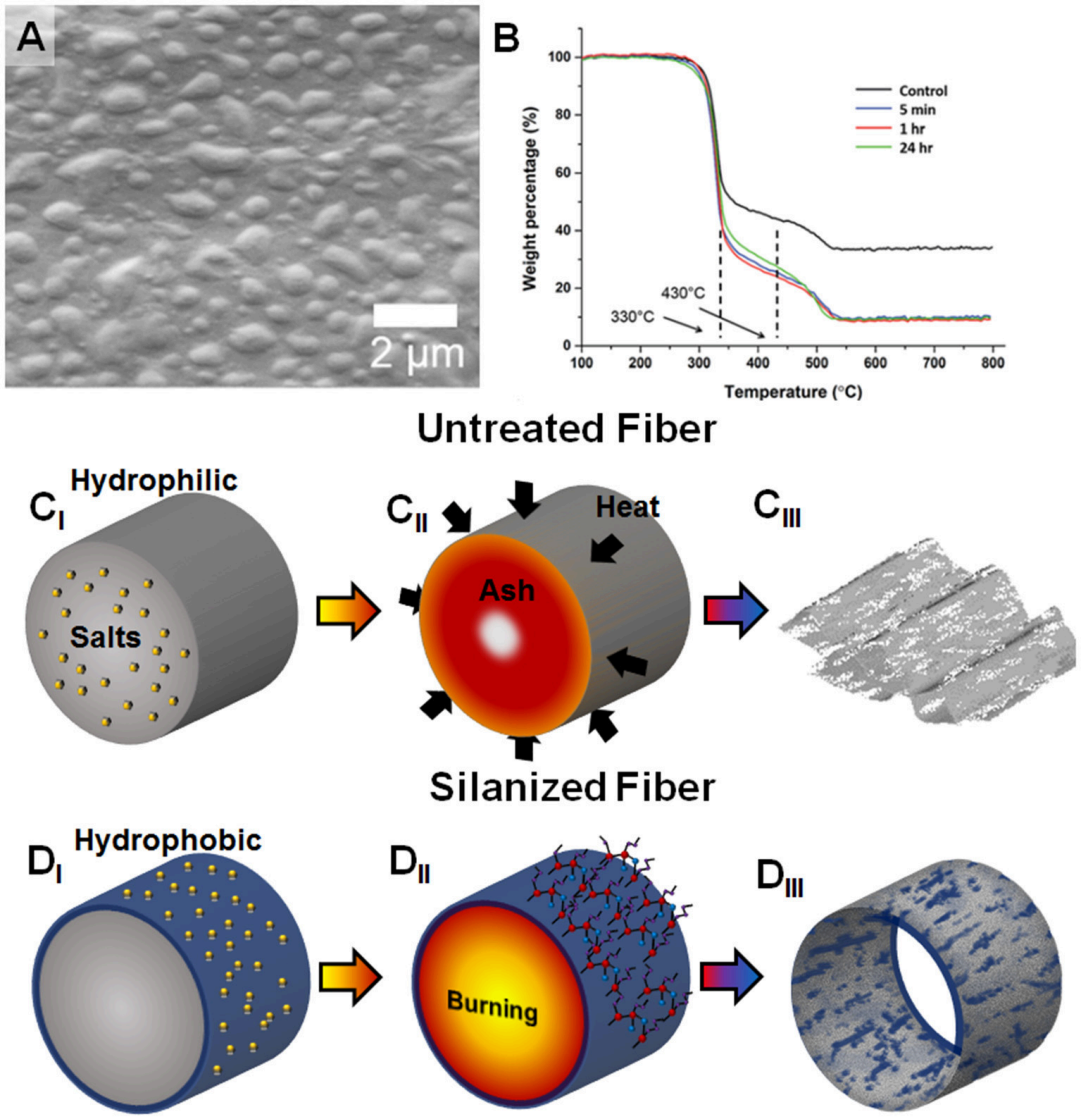
**(A)** Previous work showing generation of textured silanized amphiphobic paper surfaces (Oyola-Reynoso et al., 2016d). **(B)** Thermal degradation of silanized paper under controlled environment gives lower ash residue (Oyola-Reynoso et al., 2016a)–as published by The Royal Society of Chemistry. **(C**_I_**-C**_III_**)** Templating process for untreated cellulose fiber leads to collapsed structures, **(D**_I_**-D**_III_**)** Use of amphiphobic paper, with concomitant surface concentration of the salt and silane particles can, potentially, lead to surface concentration of the ash residues.

Utility of paper as a functional material is well demonstrated and has recently gained increased interest, in part, due to advances in controlling selective wetting (Huang and Kunitake, 2003; He et al., 2005; Sun et al., 2011; Zhou et al., 2011; Zhao and Shao, 2012; Thuo et al., 2014; Fratzl et al., 2018). Whitesides and co-workers, for example, have recently shown that noble metal nanostructures can be made using paper as a template and fuel source in a thermal-oxidative process (Christodouleas Dionysios et al., 2017). The process used is simple and involves occlusion of salts in paper followed by oxidative precipitation of metal adducts upon thermal degradation of the paper. Structures produced appears to be irregular, likely due to incomplete sintering of the metal adducts around the cellulose fibers or a manifestation of the underlying stochastic degradation process. Modifying the surface energy of cellulose could, potentially, alter produced structures by tuning the distribution of the physisorbed precursors. We Oyola-Reynoso et al. (2016a, 2017) recently demonstrated that paper treated with silanes gives tunable amphiphobicity that is characterized by hydrophobic and hydrophilic regions (Figure 1A). Reaction of paper with trichloroalkylsilanes can lead to step-growth polymerization induced self-assembly to give surface chemisorbed nano- to micro-sized polymer particles on the surface (Oyola-Reynoso et al., 2016a, 2017). The size of such generated surface particles can be varied through reaction time and temperature, which in turn leads to tunable wettability (Oyola-Reynoso et al., 2016d). This modification led to better thermal degradation (low ash) under controlled condition and without release of toxic HF (Figure 1B). The use of silane treated materials in the Whitesides approach to paper-templated synthesis could, therefore, alter both the adsorption of the salts and the degradation mechanism. We hypothesize that the silane coating would alter the morphology of templated microstructures derived from thermal degradation of paper from collapsed structures (Figure 1C) to fiber network mimicry (Figure 1D). The changes in surface energy (wettability) should promote distribution of the occluded salt at or near the fiber surface. Thus, we would expect morphological changes in the salt adducts between untreated and silanized fiber after thermal degradation (Figures 1C,D). With the surface adsorbed materials, the precipitated ashes should, in theory, closely mimic the fiber network when the paper is silanized. Presence of salt and surface silane also influences the degradation mechanism as detailed below.

Thermal degradation is triggered by ignition, which in turn depends on the concentration of the material (fuel), an oxidant (often O_2_), and activation energy. Ignition, in chemical terms, is a runaway bond dissociation/disproponation reaction cascade triggered by presence of an oxidant and thermal activation. In ambient conditions, the dissipative propagation of the ignition event is an entropically favored self-limiting set of runaway reactions commonly refered to as combusition (Schmidt-Rohr, 2015). Based on the simplified Shell ignition model for a hydrocarbon, RH, it follows that (Equation 1; Halstead et al., 1977; Sazhina et al., 1999, 2000; Sazhin et al., 1999);

(1) $$\begin{array}{r} \left[\Delta RH\right]=\frac{\Delta \left[O_{2}\right]}{\text{pm}} \end{array}$$

Where [] = concentration (molar concentration), *p* = propagation event and *m* = number of carbons in the hydrocarbon. The dependence of degradation (Δ[*RH*]) on concentration of oxygen (Equation 1) suggests that in isotropic fibrous materials ignition is expected to primarily occur on the surface of the fiber and if proper mixing and complete combustion occurs, then the fibers would degrade from surface to the core (outside-inside degradation). Where propagation is rapid, incomplete combusition will occur leading to accumulation of ash on the surface of the fiber, which in turn limits oxidant supply. It is therefore to be expected that a deposition of surface ash leads to higher residue and eventual inhibition of thermal degradation. Since the critical propagation step of the ignition is thermally activated, the dissipation—hence temperature, of the material is a critical parameter to consider in understanding degradation mechanism and eventual properties of produced ashes. According to the Shell model, changes in temperature with time upon ignition can be simplified as (Equation 2; Halstead et al., 1977; Sazhina et al., 1999, 2000; Sazhin et al., 1999);

(2) $$\frac{dT}{dt}=\frac{1}{C_{v}N_{i}}K_{p}qV\left[R^{.}\right]$$

Where *C*_*v*_ = heat capacity at constant volume (Joules/Kelvin), *N*_*i*_ = total number of molecules per unit volume—a parameter that also captures the specific surface of the material, *K*_*p*_ = ignition propagation rate (related to the concentration in each propagation step, defined in Halstead et al., 1977), *q* = exothermicity per cycle (Joules), V = volume, [*R*^.^] = concentration of the ignited hydrocarbon(s) (molar concentration). This simplified model for an isotropic fuel source captures the role of the dissipative nature of the propagating front (*q*) and the rate of propagation (*K*_*p*_), whose effect(s) on temperature is inversely proportional to the heat capacity of the material. It therefore follows that the rate of thermal degradation can be tuned either by varying concentration of material or oxidant (Equation 1) or via temperature (Equation 2).

Based on the above simplified equations, two major scenarios can be deduced *vis*: (i) based on Equation (1) as applied to fibrous materials, introduction of heterogeneity on the surface can slow or inhibit surface ignition (for example, surface grafting as in Figure 1A). In such a scenario, stochastic temperature dependent autoignition has to occur in the bulk of the material, leading to multiple propagation fronts and likely more complete combustion due to minimized local ash deposition. We (Oyola-Reynoso et al., 2016a, 2017) recently demonstrated this thermal degradation scenario under controlled environment (Figures 1A,B). (ii) From Equation (2), it follows that, in thermal degradation of heterogeneous materials, differences in specific heat capacity can provide thermal sinks/concentrators that could autoignite leading to multiple propagating fronts. In cases where one component is reactive or transforms, consumption of thermal energy at the propagating front reduces number of propagating events hence increased incomplete combustion. Based on these scenario, we hypothesized and demonstrate that interfering with thermal degradation mechanism, fibrous materials (in our case paper) can be used as templates for frugal synthesize of carbon-based materials with concomitant incorporation of oxidized adducts as demonstrated with Fe_2_O_3_. This scenario, once validated, provides orthogonal design rules to previously reported paper templated salt adduct formation.

## Materials and methods

### Materials

All reagents were obtained from Sigma-Aldrich and were used as supplied. NYX blotting paper (NYX Cosmetics), Chromatography paper (Whatman #1), and Cardstock paper (Georgia Pacific) were used as cellulose templates. These papers were selected for their variations in both fiber density and types of fillers. (See Supporting Information, Figure S1).

### Salt solution

Iron (III) Chloride (FeCl_3_, 97% Sigma-Aldrich) salt solution (10 g/L, 0.06 M) was prepared using ultrapure water (obtained from Thermofisher Smart2Pure6 water purification system) and used as prepared to impregnate the paper.

### Paper preparation

Paper samples (NYX Blotting, Whatman No. 1 Chromatography, Georgia-Pacific Cardstock) were cut into 4 × 4 cm squares. Surface modification was performed by vapor phase deposition as previously described (Oyola-Reynoso et al., 2016d). The process was conducted in a pre-heated desiccator containing drierite. Paper samples were placed in the desiccator with 100 μL of trichloro(1H,1H,2H,2H-perflurooctyl)silane and immediately evacuated for *ca*. 2 min. The evacuated desiccator was placed in an oven maintained at 95°C and reaction allowed to run *in vacuo* for 1 h.

### Saturation of paper in salt

All paper samples were pre-weighed before saturation with the salt. Paper samples were submerged in the solution for 10–15 s followed by drying in an oven at 50°C for *ca*. 7 min. Once dried, the samples were re-weighed, and the process repeated until there was no observable change in mass.

### Degradation

Salt saturated paper were thermally degraded via two methods: (I) Samples were ignited in a fume hood with a butane lighter and allowed to burn (flash burning), (II) thermal degradation was performed using thermogravimetric analysis (TGA) equipment (TA Instruments Q50) from room temperature to 800°C (20°C/min, air/N_2_ purge gas). Residual ashes were then characterized using SEM and EDS.

### Scanning electron microscopy (SEM) and energy dispersive X-ray spectroscopy (EDS)

Fabricated shapes were characterized and imaged by Scanning Electron Microscopy (FEI Quanta 250 FEI-SEM). The SEM was operated under high vacuum with accelerating voltage of 2–3 kV at 10.5 mm working distance. Everhart-Thorley Secondary electron detector was used to take micrographs. Line integration settings was used to ensure high signal to noise ratio at lower beam currents. Elemental composition was determined using EDS with a silicon drift detector at an increased accelerating voltage of 10 kV for increased count rate. EDS Map analysis was done with 256 × 256 pixels resolution, 3 frames, 100 μs dwell time setting using the AztecOne software on the same instrument.

## Results and discussion

Three types of paper, namely chromatography (CH), card stock (CS) and blotting (NYX) paper were used in this work. The papers were chosen based on variation in fiber density, grammage (NYX>CS>CH) and filler content (no fillers in CH, precipated calcium carbonate in CS–expected to react with the by-product of silanization and talc in NYX–unreactive). The salt, FeCl_3_, was used as the model metal ion source due to ease of access and differences in color with changes in oxidation state and potential to form magnetic precipitates.

**Figure 2:**
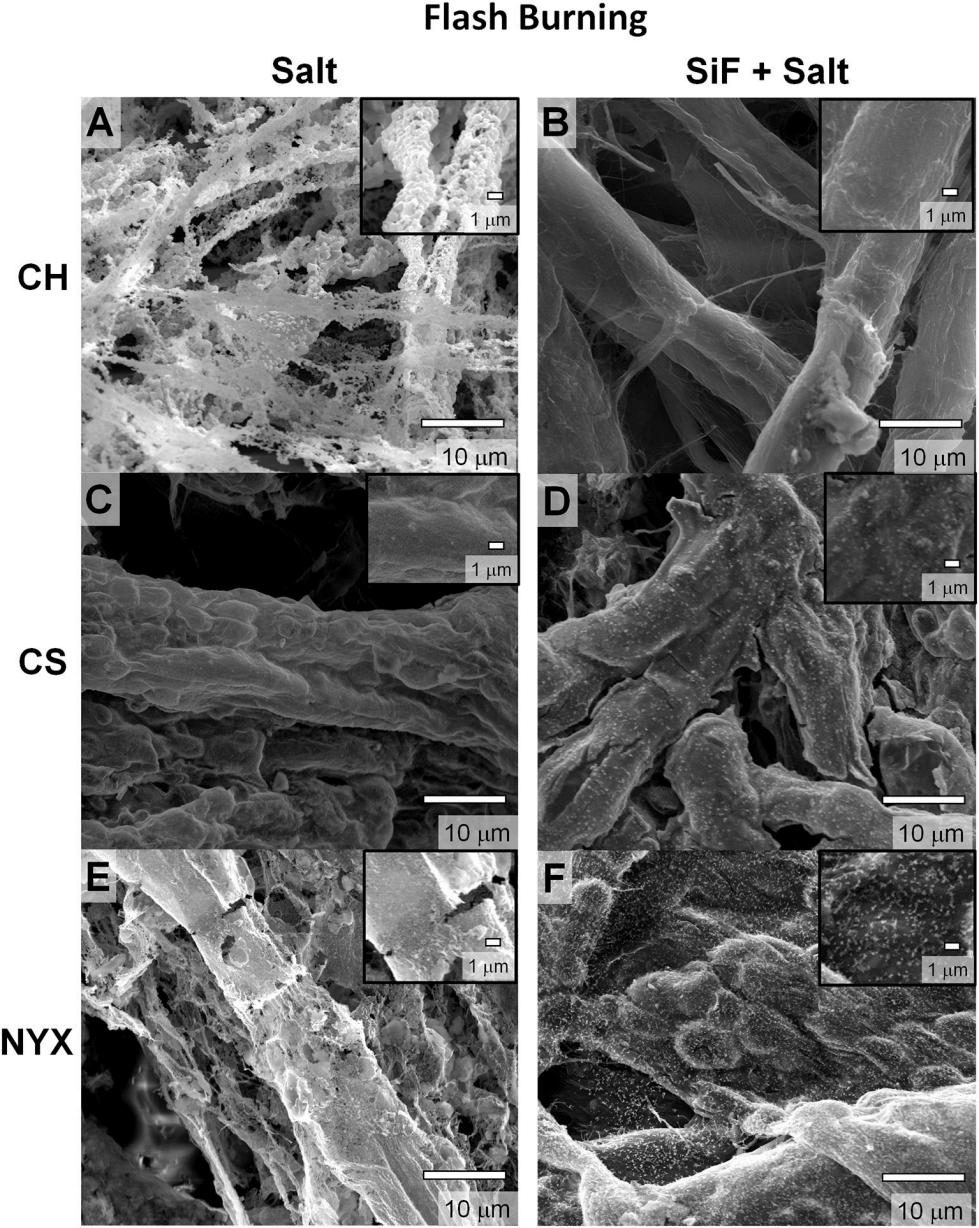
Scanning electron microscopy (SEM) images of templated structures derived from pristine (salt) and silanized (SiF+salt) cellulose respectively burned by flash thermolysis. **(A,B)** Chromatography paper (CH). **(C,D)** Cardstock paper (CS). **(E,F)** Blotting paper (NYX).

Paper loaded with the salt, as expected, changes color with significant differences observed with increase in fiber densities and treatment times. As explained above, we employed two main approaches to thermal degradation; (i) direct flash pyrolysis- where the paper was ignited into an open flame, (ii) controlled degradation- where the paper was thermally degraded under controlled environment and heating rates. Under flash thermolysis, the impregnated samples burned with a bright yellow flame. As expected, when untreated paper samples (with or without salt , see Supporting Information Figure S2) were degraded, a complete collapse of the fibers occurred precipitating the ash along the now degraded network (Figure 2A). As previously shown by Whitesides and co-workers (Christodouleas Dionysios et al., 2017), with the untreated paper, the salt sinters and adopts a connected porous network akin to that of the paper fiber network (Figure 2A).

### Effect of paper fillers

We compared thermal degradation of papers with (CS and NYX) and without (CH) fillers after impregranation with Fe salt (Figure 2). We observe that the filler-free paper largely degrades to form porous structures (Figure 2A). These structures are fragile and most of the paper fiber has been fully degraded. On the other hand, CS paper contains PCC (precipitated CaCO_3_) that can release CO_2_ under thermal degradation or convert to CaCl_2_ on reaction with HCl (a by-product of the silanization with trihalo alkylsilane). Produced CO_2_ retards thermolysis hence likely to promote incomplete combustion by slowing the propagation of the ignition event (low *K*_*p*_ in Equation 2). Presence of amorphous carbon and oxidized Fe adducts may lead to increased residual ashes. As a result, the structure of the paper fibers is better preserved and show better continuity compared to CH paper (Figure 2C). Similarly, for the NYX paper that contains a non-reactive filler and has higher grammage, the native fiber network is more evident than in the CH paper, although a significant number of defects on the residue were observed compared to the CS. We can therefore infer that the nature of the residual ashes depends on amount of fiber per unit area (Equation 1) and control over the rate of ignition propagation, *K*_*p*_, (combustion) process.

### Effect of fiber density

For comparison purposes, we investigated papers with increasing density, CH < CS < NYX, as previously reported (Oyola-Reynoso et al., 2016d). The NYX paper is ~2x the fiber density relative to CH and contains talc (Mg/Al oxide) fillers that do not reactively interfere with the degradation process. We observe that high density papers produced more residues as predicted by Equations (1, 2). The NYX paper, for example, produces significantly more carbonaceous mass than the CH especially without surface treatment (Figures 2A vs. 2E). Although this observation is in line with the predictions based on propagation of the ignition event, we exercise caution in its over-interpretation as the talc filler may play a yet to be determined role. We therefore infer an association, but not a causation, between residual mass and density of the paper.

### Modifying surface energy as a design tool for creating structures

**Figure 3:**
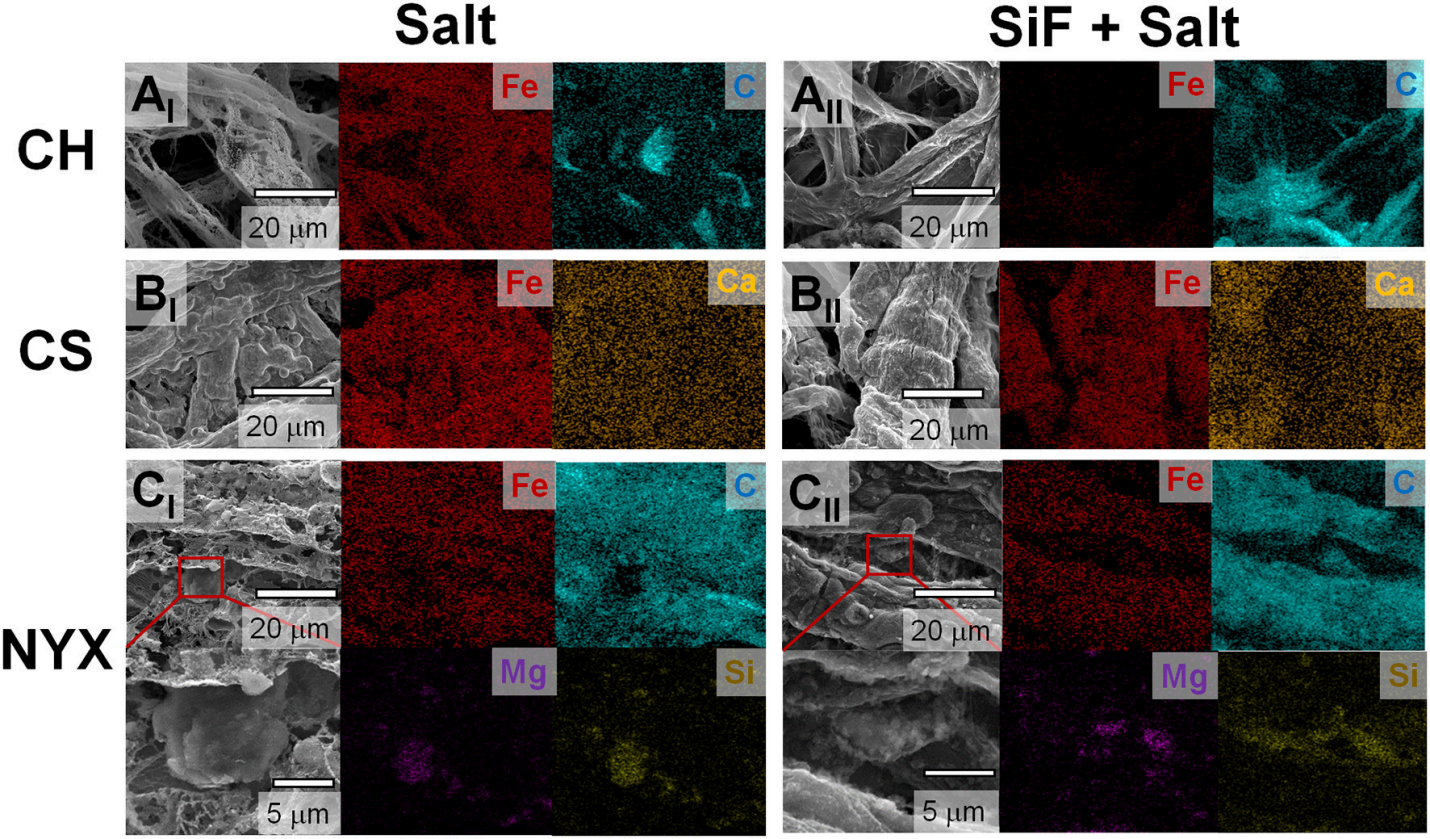
Distribution maps of key elements in the residual ash from flash burning as observed by Energy Dispersive X-ray spectroscopy (EDS) of the structures derived from hydrophilic paper treated with (I) Salt and (II) SiF + Salt. **(A)** Chromatography paper. **(B)** Cardstock paper. **(C)** Blotting paper.

Occlusion of salt (higher thermal coefficient than cellulose) on paper fibers significantly improves heat transfer into the material, which in turn affects thermal degradation rate. The modification of surface energy by silane treatment leads to omniphobicity which in turns tunes liquid absorption into the paper. Controlled solvation in turns tunes amount of salt adsorption/occluded in the paper fibers. We previously demonstrated that, silanized (R^F^) paper biodegrades slower than pristine paper, in part, due to slow absorption of water (Oyola-Reynoso et al., 2016b,c). We infer that thermal degradation of paper fibers can, therefore, be tuned through silanization, which concomitantly affects the resulting structure of the residues. This hypothesis has been confirmed as shown in Figure 2 above (i.e., Figures 2A,C,E vs. 2B, D,E). Besides the effect of treatment on structure, we also observe changes in the overall composition of the ashes. Considering the low-fiber density, filler-free, CH paper, a clear difference in morphology of the residues is observed (Figures 2A vs. Figure 2B) with concomitant treatment-dependent difference in distribution of residual carbon (Figures 3A_I_-**A**_II_). In the case of CS, most significant difference was observed in the distribution of the filler adducts. The untreated CS paper showed a uniform distribution of Ca (Figure 3B_I_) while upon silanization asymmetric distribution is observed with most of the Ca deposition away from the residual carbon and Fe (Figure 3B_II_). Enhanced residual carbon concentration was also observed with treatment for NYX, however, in this case the distribution of the filler adduct did not depend on treatment. (Figures 3C_I_-C_II_) Comparing filler adducts in CS (Figure 3B) and NYX (Figure 3C), we infer that when fillers are transformed during the processing, translation (movement) may occur leading to significant reorganization. Based on the high overlap in distribution of the salt and residual carbon, (Figure 3 and Supporting Information Figure S3) we infer that when the salt is occluded into the fibers (as opposed to physisorbed on the fiber), then localization of the ignition event is more likely leading to better patterning of residual ashes along the native fiber network.

### Effect of heating rate

**Figure 4:**
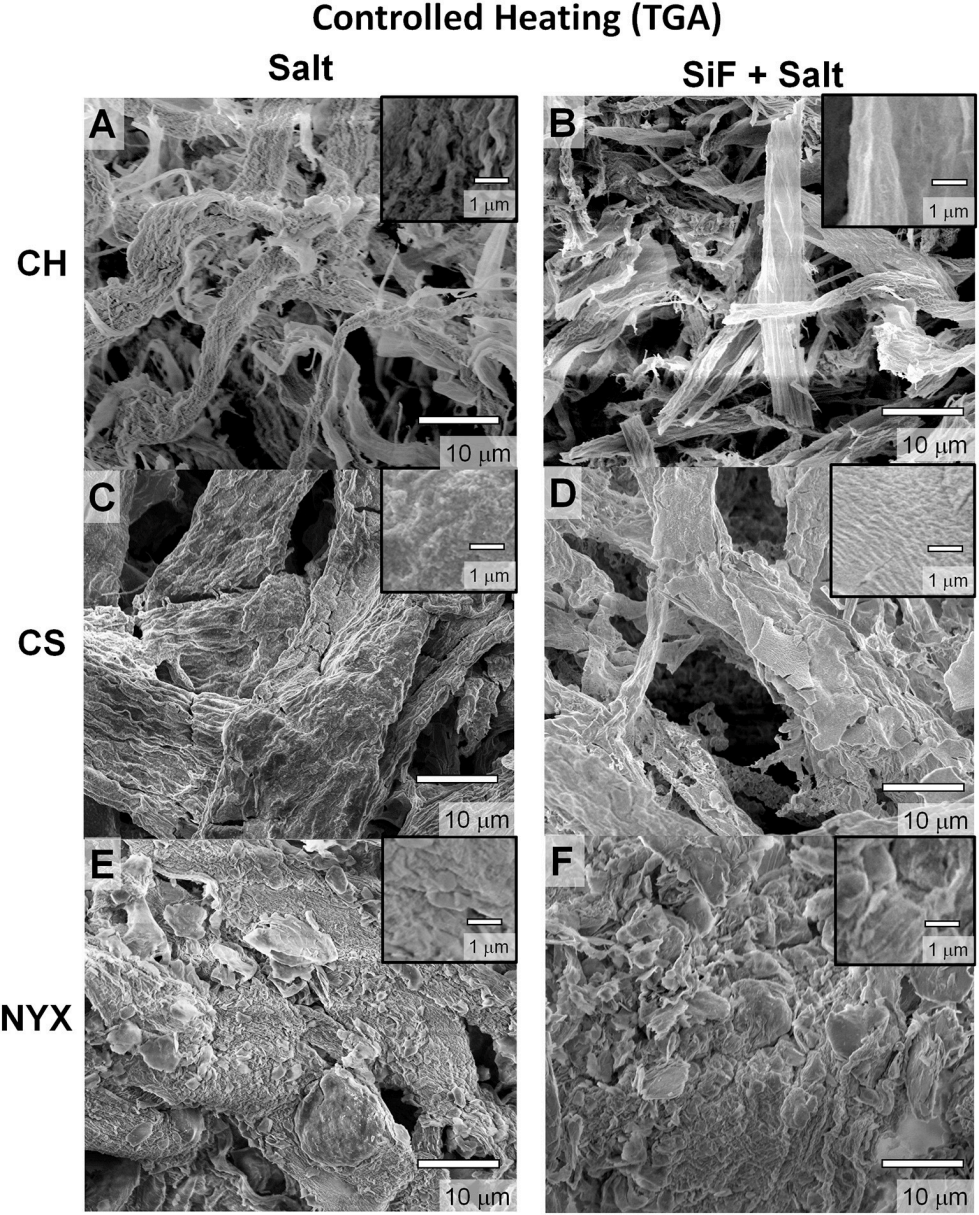
Scanning electron microscopy (SEM) images of templated structures derived from pristine (salt) and silanized (salt+SiF) cellulose respectively degraded in a TGA oven (controlled burning). **(A,B)** Chromatography paper (CH). **(C,D)** Cardstock paper (CS). **(E,F)** Blotting paper (NYX).

To assess the effect of heating rate, we compared flash burning with controlled indirect heating (performed in a TGA instrument) under air (80% N_2_, 20% O_2_). Figure 4 shows SEM images of the samples after burning in TGA up to 800°C (at a heating rate of 20°C per min). From direct comparison with flash burning (open flame), we observe an enhanced deposition of ash along the fiber network (Figure S2) albeit with significant collapse of individual structures. We infer that rapid but incomplete degradation under flash burn leads to deposition of residual carbon hence enhanced fiber structure integrity. Due to requisite airflow in a TGA oven, effect of high oxidant concentration likely affects the ignition point and eventual propagation hence the extent of fuel (paper) consumption. We confirm compositional differences via SEM-EDS (Figure S5 and Table S1). Carbon content in flash heated samples are significantly higher (except for CS , see Supporting Information Figure S4 and Table S1 for distribution ratios of different elements), supporting the inference that there is likely incomplete burning of the cellulose fibers. We hypothesize that in CS, the reactive PCC fillers affect degradation rate orthogonal to what is under flash burning, in part due to gaseous exudates promoting local mixing hence improved combustion. A secondary effect could be due to thermal energy accumulation in the PCC prior to degradation leading to multiple ignition events. We, however, cannot decouple these two processes but can infer that a reactive filler will interfere with the degradation process, hence the product, irrespective of the pathway.

**Figure 5:**
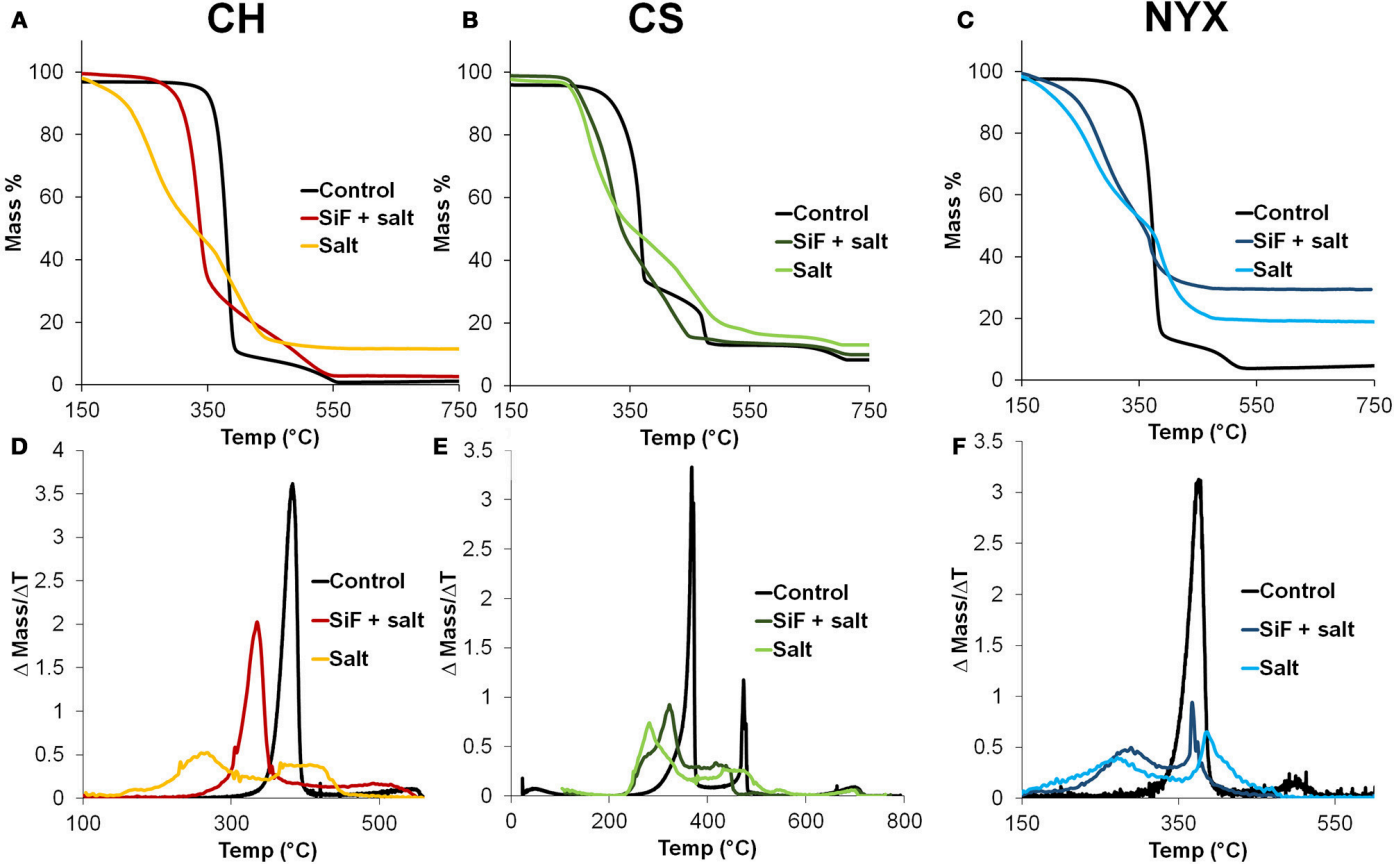
**(A–C)** Thermogravimetric analysis (TGA) of templated pristine and silanized cellulose. **(D–F)** Associated differential TGA highlighting the degradation maxima and number of degradation events.

Analysis of the rate of degration from the TGA reveals that all papers degrade via multiple steps in a manner that depends on the nature of the paper and presence or absence of the surface modifier. Figures 5A–C shows the TGA plots for all samples (control, salt and SiF+salt). Control samples all follow similar degradation paths with two main events, one large and quick and a second shorter and slow event. We Oyola-Reynoso et al. (2016a) previously reported similar degradation patterns for the NYX paper under controlled oxygen (Figure 1B). For clarity, we represent the thermal degradation steps through the differentiated curves (change of mass with change in time) such that each degradation step is represented by a peak (Figures 5D–F). Baseline resolution of the peaks indicates two clearly decoupled events, where one comes to completion prior to the following step. We observe baseline resolution for the control samples, especially in presence of fillers (Figures 5E,F). Degradation of control paper samples is dominated by the first step occurring at *ca*. 350°C. In presence of PCC, a second degradation event occurs at 450°C but at ~500°C in presence of talc. To further support our inference that the predominance of the second peak is likely due to the presence of the fillers, we note that upon occluding salt into the papers, two distinct peaks were observed. The first peak initiated significantly earlier than in the control sample, further supporting our hypothesis that salts, with higher thermal coefficient, can act as heat concentrators promoting local ignition. The second peak also initiates at a low temperature than in the control although the intensity shows a dependence on the nature of the filler. The significantly different mass loss in-between the first and second peak in presence of the salt, however, further supports the idea that, under flash burning, physisorbed materials can interfere with the propagation of the ignition event, hence, promote incomplete combustion. From Equations (1, 2), we caution that this being a kinetically driven event, the patterns are highly dependent on oxygen concentration and its flux into the burning material. In fact, comparing current NYX control data (Figure 5F) with previously reports for degraded under reduced oxygen (Figure 1B), we observe slight differences in the initiation temperatures and residue mass. From the previous study, however, we observe that the initiation temperature is slightly lower upon silinazation supporting the observations above. When salt is occluded in silanized papers, however, hybrid behavior is observed. The arrangement of the degradation peaks are akin to a hybrid degration of the control samples and those with occluded salts. We infer that silanization limits occlusion of the salt hence a smaller mole fraction of the salt is physisorbed. The shifting of degradation peaks, therefore, are dependent on salt concentration in the paper which can be tuned through surface energy (wettability) modification.

## Conclusion

Tuning the thermal degradation process, either through chemi- and/or physi-sorbed adducts on paper, affects the propagation of the ignition event leading to enhanced incomplete combustion. This reduced degradation process precipitates structured networks akin to the native fiber structure. We can therefore infer that modification of paper with concomitant felicitous choice of degradation conditions is a viable pathway to synthesize networks of metallic adducts. Tunability of the paper network during pulp processing can therefore be exploited in a tunable thermal degradation process to produce carbon networks impregnated with various metal-salt adducts.

## Author contributions

J-FB and MT conceived the idea and directed the work. PG, SO-R, AM, and BC run the experiments, collected data, analyzed samples. All authors contributed in data analysis and in writing the manuscript.

### Conflict of interest statement

The authors declare that the research was conducted in the absence of any commercial or financial relationships that could be construed as a potential conflict of interest.
