# Supplementary material for: Inverting Thermal Degradation (iTD) of Paper Using Chemi- and Physi-Sorbed Modifiers for Templated Material Synthesis
Source: Front Chem. 2018 Sep 7;6:338. doi: 10.3389/fchem.2018.00338 (PMC6137831; doi:10.3389/fchem.2018.00338)
Supplement: Supplementary file 1 [file Data_Sheet_1.pdf]

Supplementary Material

Inverting Thermal Degradation (^{i}TD) of Paper Using Chemi- and Physi-Sorbed Modifiers for Templated Material Synthesis

Paul R. Gregory[1#], Andrew Martin[1#], Boyce S. Chang[1], Stephanie Oyola-Reynoso[1], Jean-Francis Bloch[2*], Martin M. Thuo[1*]

[1]Department of Materials Science & Engineering, Iowa State University, Ames, IA, USA
[2]Univ. Grenoble Alpes, CNRS, Grenoble INP†, Institute of Engineering, 3SR, F-38000 Grenoble, France

[#]Equal contribution

*** Correspondence:**
Prof. Martin M. Thuo, mthuo@iastate.edu
Prof. Jean-Francis Bloch, jean-francis.bloch@pagora.grenoble-inp.fr

Keywords: cellulose, nanostructures, salt, FeCl3, surface polymerization, heat treatment

Supplementary Figures

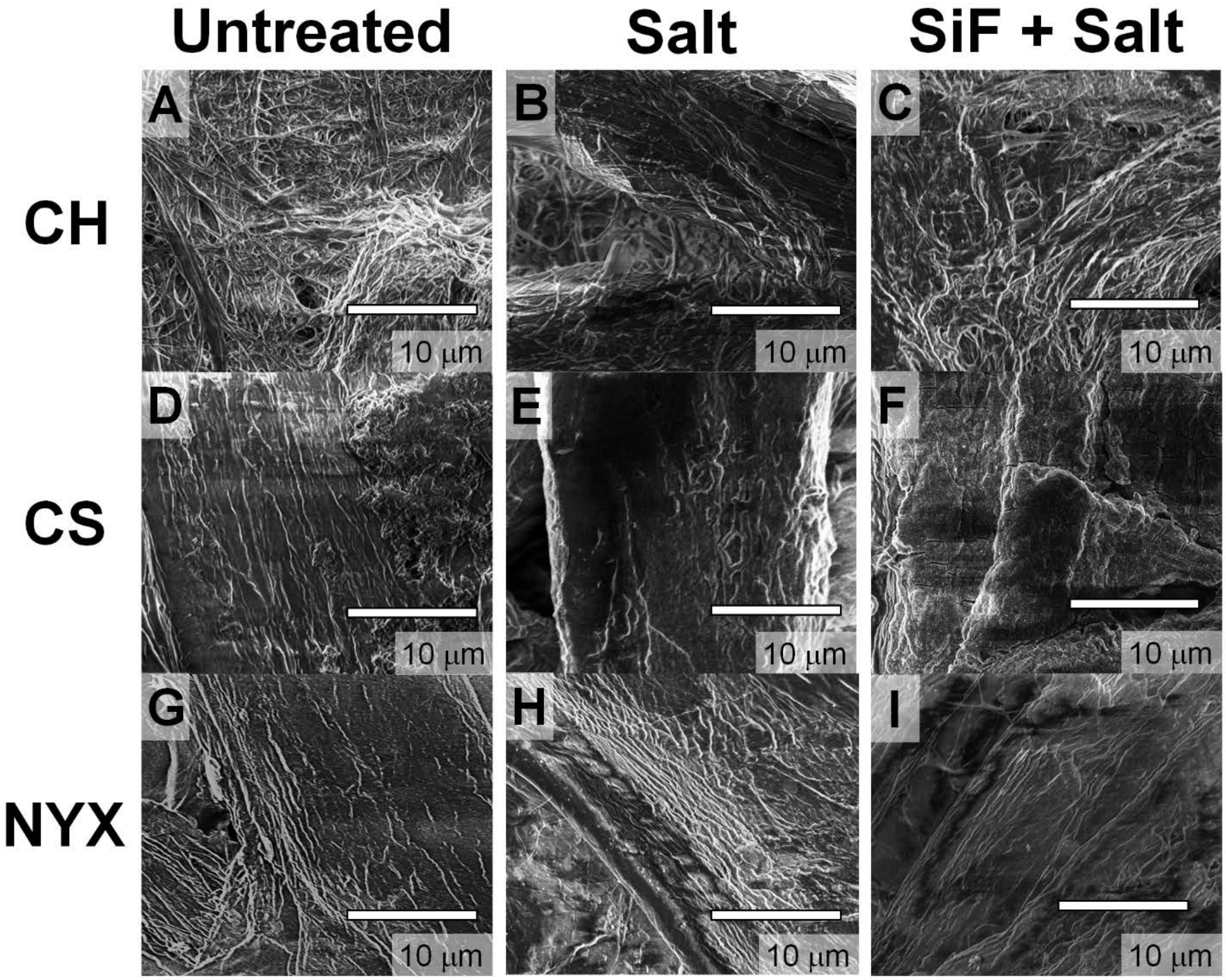

Supplementary Figure S1. Micrographs of unburned samples for each treatment (untreated, salted, silanized respectively). (A-C) Chromatography paper. (D-F) Cardstock paper. (G-I) Blotting paper.

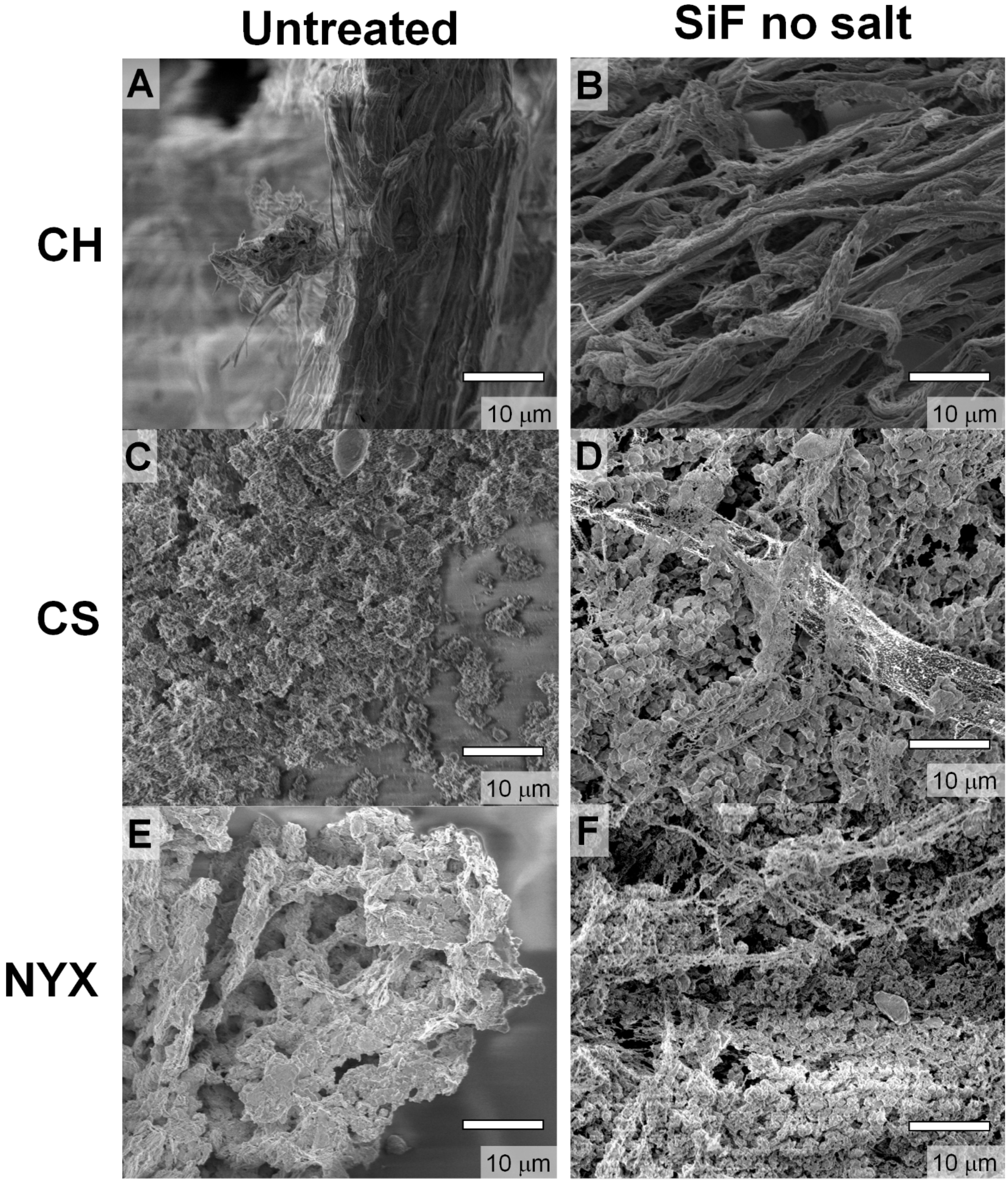

Supplementary Figure S2. Micrographs of burned samples for control samples (untreated) and silanized without salt. (A-B) Chromatography paper. (C-D) Cardstock paper. (E-F) Blotting paper.

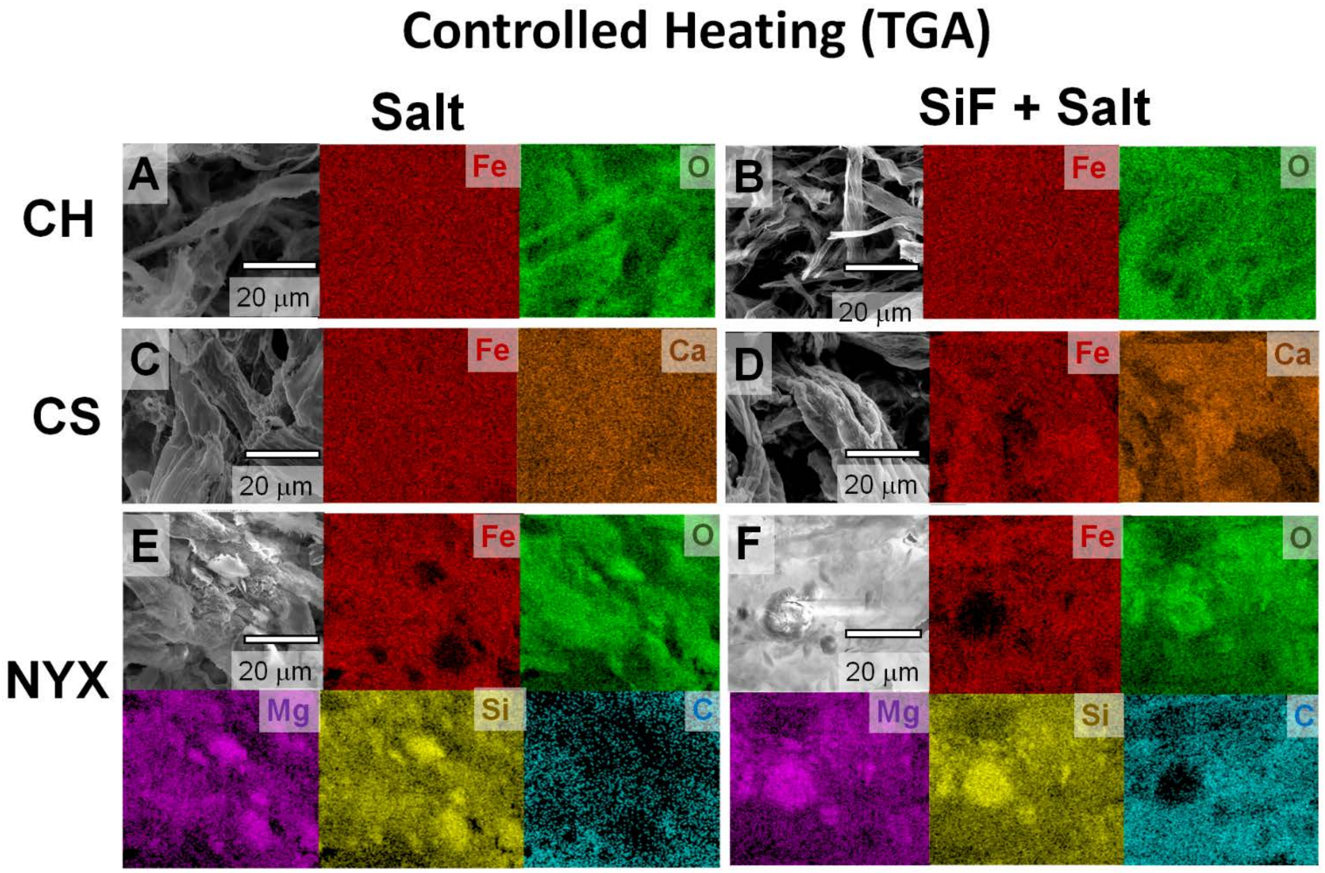

Supplementary Figure S3. Energy dispersive X-ray spectroscopy (EDS) of samples burned in TGA. (A-B) Chromatography paper. (C-D) Cardstock paper. (E-F) Blotting paper.

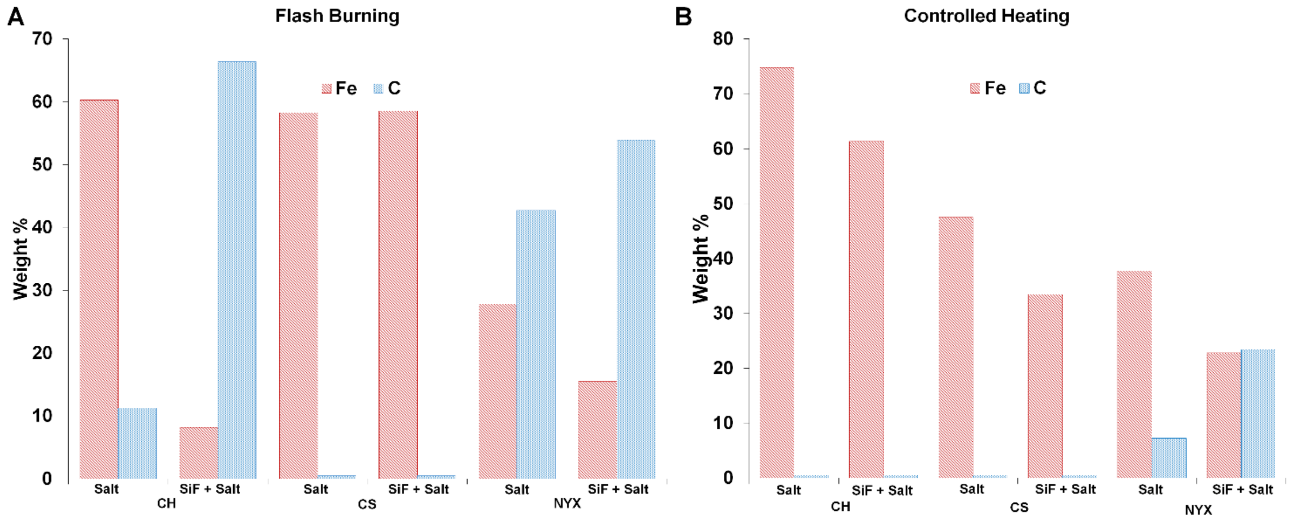

Supplementary Figure S4. Weight percent of Fe and C of the heat-treated samples for (A) flash burning and (B) controlled heating. Data values are single points.

Supplementary Table S1. Amount of iron and carbon leftover in paper ashes after burning.

Paper	Treatment	Element	Flash Burn (wt%)	Controlled Heat (wt%)
**CH**	**Salt**	Fe	60.27	74.8
		C	11.26	Trace
	**SiF + salt**	Fe	8.15	61.36
		C	66.4	Trace
**CS**	**Salt**	Fe	58.27	47.49
		C	Trace	Trace
	**SiF + salt**	Fe	58.53	33.45
		C	Trace	Trace
**NYX**	**Salt**	Fe	27.82	37.72
		C	42.72	7.23
	**SiF + salt**	Fe	15.48	22.83
		C	53.89	23.4
